# Predictors for sentinel lymph node mapping failure using indocyanine green injection in apparent early stages of endometrial cancer: A single‐center prospective study

**DOI:** 10.1002/ijgo.70123

**Published:** 2025-04-10

**Authors:** Petra Bretová, Luboš Minář, Petra Ovesná, Vít Weinberger, Michal Felsinger, Michaela Koblížková, Jitka Hausnerová, Eva Jandáková, Tatiana Stupková

**Affiliations:** ^1^ Department of Gynecology and Obstetrics University Hospital Brno Brno Czech Republic; ^2^ Faculty of Medicine Masaryk University Brno Czech Republic; ^3^ Faculty of Medicine, Institute of Biostatistics and Analyses Masaryk University Brno Czech Republic; ^4^ Department of Pathology University Hospital Brno Brno Czech Republic

**Keywords:** biopsy, endometrial cancer, myomas, obesity, sentinel lymph node

## Abstract

**Objective:**

The current study aimed to analyze predictive factors of sentinel lymph node mapping failure in apparently early stages of endometrial cancer using intracervical indocyanine green injection.

**Methods:**

A single‐center prospective study was conducted between June 2019 and August 2023 at the Department of Gynecology and Obstetrics, University Hospital Brno, Czech Republic. All patients with apparently early stage (I or II according to FIGO [International Federation of Gynecology & Obstetrics] 2009) endometrial cancer, who were indicated for sentinel node biopsy were consecutively included. The injection of 4–6 mL of indocyanine green was applied superficially and deeply into cervical tissue at the 3‐ and 9‐o'clock positions. Patients' clinical data, surgical characteristics, and histopathological information were recorded. Univariable and multivariable regression analyses were applied.

**Results:**

A total of 225 patients were eligible during the study period. Considering bilateral and unilateral failed mapping together, the only statistically significant factors for risk of failure in univariable analysis were body mass index (BMI; *P* = 0.036), FIGO 2009 stage (*P* = 0.019), and the presence of a myoma (*P* = 0.017). Nevertheless, when the multivariable logistic regression analysis was applied, all factors became statistically insignificant except for myoma (*P* = 0.031). Regarding only bilateral mapping failure, in univariable analysis, BMI (*P* = 0.021) and FIGO 2009 stage (*P* = 0.046) were significant predictors of failure. Interestingly, multivariable logistic regression analysis revealed that in addition to BMI (*P* = 0.007), age (*P* = 0.004) was also an independent predictor of bilateral failure.

**Conclusions:**

Higher BMI and age were statistically significant independent factors for bilateral sentinel node mapping failure in early‐stage endometrial cancer.

## 1 INTRODUCTION

Sentinel node biopsy (SNB) has become the leading lymph node surgical staging method in endometrial cancer (EC) in recent years. Initially, only low‐risk and intermediate‐risk patients underwent SNB; however, according to the guidelines of the European Society of Gynecological Oncology (ESGO), the European Society for Radiotherapy and Oncology (ESTRO), and the European Society of Pathology (ESP), SNB is now an acceptable alternative to systematic lymphadenectomy, even for women with high‐intermediate and high‐risk early stage (I or II) EC.^1^ Furthermore, according to ESGO quality indicators for the surgical treatment of EC, SNBs should be performed in 90% of patients undergoing lymph node staging.^2^ Side‐specific pelvic lymphadenectomy should be provided in cases of SNB failure, and debulking of enlarged lymph nodes is performed regardless of mapping.^1^

In addition to reducing postoperative morbidity associated with systematic lymphadenectomy,^3^ SNB allows more precise pathological assessment and detection of low‐volume metastases: micrometastasis and isolated tumor cells (ITCs), which could be missed by standard histological evaluation of multiple lymph nodes.^4^

The use of indocyanine green (ICG) has significantly improved the accuracy of sentinel lymph node (SLN) detection, supplementing the traditionally used technetium (Tc99) and blue dyes. Bilateral detection using intracervical injection of ICG achieves a 78%–85% success rate.^5^, ^6^, ^7^, ^8^

The importance of successful bilateral SLN mapping is indisputable; however, there are still only a few studies focusing on risk factors of SNB failure in EC using intracervical ICG injection. Understanding these factors is crucial for surgerical planning strategies. A recent meta‐analysis by Raffone et al. included only six studies.^9^ They identified ICG <3 mL, FIGO (International Federation of Gynecology & Obstetrics) stage III or IV, enlarged lymph nodes, and lymph node involvement as significant predictive factors of SLN mapping failure.

As SNB is now the leading surgical staging method, we believe it is important to understand the main limitations of the procedure. Our study aimed to analyze predictive factors of SLN mapping failure in apparently early stages of EC using intracervical ICG injection while excluding already known risk factors, which could be eliminated (enlarged lymph nodes, advanced disease, ICG <3 mL). We analyzed both bilateral and unilateral mapping failure to determine whether there are factors that might have a local or systemic impact on SLN detection success.

## 2 MATERIALS AND METHODS

### 2.1 Patient cohort and data collection

**FIGURE 1:**
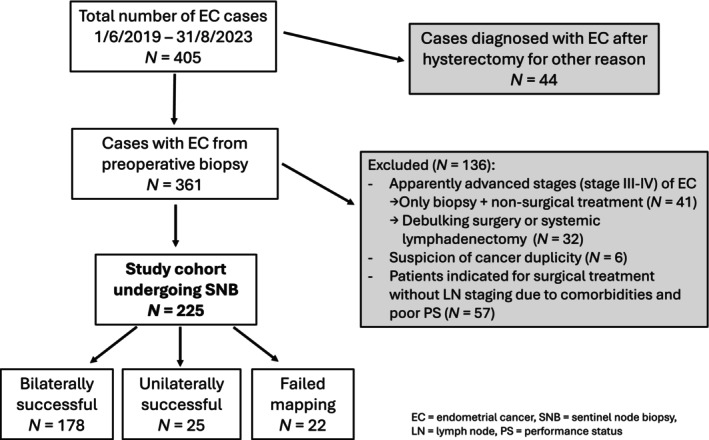
Flowchart describing the study population. EC, endometrial cancer; LN, lymph node; PS, performance status; SNB, sentinel node biopsy.

The prospective study took place at the Department of Gynecology and Obstetrics, University Hospital Brno between June 2019 and August 2023. All patients with apparently early stage of EC (FIGO stage I or II) indicated by the Multidisciplinary Oncogynecologic Tumor Board of University Hospital Brno for surgical treatment with SNB (according to ESGO/ESTRO/ESP^1^ recommendations) were consecutively included. Patients with suspicion of advanced disease (FIGO stage III or IV), cancer duplicity, and those deemed unsuitable for surgical treatment were excluded (Figure 1). Data were collected from an institutional database and surgical and histological reports. Informed consent to use their clinical and histopathological data was obtained from all patients included in the study. The study was approved by the University Hospital Brno Ethics Committee (approval number 02‐190122/EK).

Patient characteristics included age, body mass index (BMI), previous cervical and pelvic surgery (cone biopsy, myomectomy, cesarean section, adnexal surgery, retroperitoneal surgery), previous pelvic radiotherapy, and previous chemotherapy. Surgical characteristics recorded were approach (laparoscopic/open), adhesiolysis, length of surgery, blood loss, SLN mapping success (bilateral/unilateral/failure), and surgical and postoperative complications (perioperative bleeding, large vessels/nervous/ureter injury, postoperative lymphocyst/lymphoedema). Histopathological data reported were tumor histotype and grade, molecular classification, myometrial infiltration, cervical involvement, tumor localization and size, FIGO 2009 stage, lymphovascular space invasion (LVSI) status (absence/focal/substantial), microcystic, elongated, and fragmented (MELF) type of invasion, uterine length, presence of myomas/adenomyosis, number of SLN removed, and SLN status (negative/macrometastasis/micrometastasis/ITC).

### 2.2 Preoperative staging

The preoperative staging procedure in histologically proven EC contained a gynecological clinical examination, transvaginal and transabdominal gynecologic ultrasound performed by expert ultrasonographers, and a chest‐abdominal‐pelvis computed tomography scan. Preoperative procedures focused on both the local spread of the disease and discovering lymph node metastases or distant metastases, which would be a criterion for the patient's exclusion from the study.

### 2.3 Surgical procedure

Surgeries were performed by two groups of surgeons with different levels of experience. Advanced surgeons were defined as certified gynecologic oncologists with years of surgical experience, while trainees were fellows in gynecologic oncology working under the supervision of advanced surgeons. All of the surgeons followed the same protocol: 4–6 mL of ICG (1.25 mg/mL) were injected intracervically immediately before surgery in an operating theater. We used the technique of superficial (3–5 mm) and deep (10–20 mm) application into cervical tissue at the 3‐ and 9‐o'clock positions. In the situation of no cervical involvement, a uterine manipulator was inserted. Sentinel nodes were searched in the retroperitoneum using the Pinpoint Fluorescence Imaging System (Novadaq) endoscopically or by portable handheld imager in case of primary open surgery or conversion into laparotomy. Removed SLNs were sent to definitive histopathological ultrastaging without perioperative frozen section. Reinjection of ICG was performed in cases of fluorescent signal absence in the retroperitoneal space. The surgery continued with standard extrafascial hysterectomy and bilateral salpingo‐oophorectomy. Any macroscopically enlarged nodes (>2 cm) were removed as part of debulking regardless of mapping. Complete or side‐specific pelvic lymph node dissection was added in cases of mapping failure, providing the patient had preoperatively high‐intermediate or high‐risk disease according to ESGO/ESTRO/ESP guidelines.^1^

### 2.4 Histopathological evaluation

All SLNs were fixed in 10% buffered formalin, sliced at 2‐mm lamellas, embedded in paraffin, and further examined by ultrastaging protocol. This protocol consists of 4‐μm‐thick consecutive sections: two stained with hematoxylin–eosin and cytokeratins (AE1/AE3), followed by two additional sections stained with hematoxylin–eosin obtained at regular 200‐μm intervals. This sequence of sections continued until there was no lymph node tissue left. We classified lymph nodes as follows: negative, ITCs (≤0.2 mm or single cells/clusters of cells ≤200 cells in a single SLN cross‐section), micrometastasis (0.2–2 mm), and macrometastasis (>2 mm). Histopathological examination of tumor tissue and molecular testing were performed following national and international guidelines.^1^, ^10^

### 2.5 Statistical analysis

Standard descriptive statistics were used to describe the cohort: mean, standard deviation (SD), median, and interquartile range (IQR) for continuous variables, and absolute and relative frequencies for categorical variables. Comparisons of variables between the two groups (bilateral success vs. any failure, and bilateral failure vs. any success, respectively) were performed using the Mann–Whitney test and Fisher test, respectively.

Univariable and then multivariable logistic models were created to estimate the risk of SNB mapping failure. Any of the covariates that were statistically significant in the univariable model (*P* < 0.10) and clinically important factors were initially considered for inclusion in the multivariable model. Variable selection was performed using a backward stepwise approach based on Akaike Information Criterion minimization to optimize model fit while avoiding overfitting. The risk of failure was quantified by odds ratios (ORs) with 95% confidence intervals (CIs). Any failure (bilateral or unilateral) and exclusively bilateral failure were modeled. Analyses were performed in R software version 4.3.2 (R Foundation for Statistical Computing), models were conducted using the lme4 package. The significance level was set at 5%.

## 3 RESULTS

During the study period, 405 patients were diagnosed with EC at our institution. Ultimately, a total of 225 patients met the eligibility criteria (Figure 1). The median patient age was 65 years, and BMI was 32 kg/m^2^ (Table 1).

**TABLE 1:** Clinical and histopathological characteristics.

Characteristic	Overall, *N* = 225	Bilateral success, *N* = 178	Failure, *N* = 47
Age (years)			
Mean (SD)	65 (10)	64 (10)	66 (9)
Median (IQR)	65 (58–72)	65 (58–72)	65 (61–73)
BMI			
Mean (SD)	32 (6)	31 (6)	34 (7)
Median (IQR)	32 (28–36)	31 (27–36)	33 (29–37)
Cone biopsy	13 (100.0%)	11 (84.6%)	2 (15.4%)
Myomectomy	2 (100.0%)	2 (100.0%)	0 (0.0%)
Cesarean section	24 (100.0%)	17 (70.8%)	7 (29.2%)
Adnexal surgery	18 (100.0%)	14 (77.8%)	4 (22.2%)
Retroperitoneal surgery	0 (NA)	0 (NA%)	0 (NA)
Previous pelvic radiotherapy	0 (NA)	0 (NA%)	0 (NA)
Previous chemotherapy	3 (100.0%)	2 (66.7%)	1 (33.3%)
Histotype			
Endometrioid (including mucinous)	211 (100.0%)	168 (79.6%)	43 (20.4%)
Nonendometrioid	14 (100.0%)	10 (71.4%)	4 (28.6%)
Grade			
Low grade (grade 1 or 2)	189 (100.0%)	149 (78.8%)	40 (21.2%)
High grade (grade 3)	36 (100.0%)	29 (80.6%)	7 (19.4%)
Myometrial infiltration			
<50%	173 (100.0%)	136 (78.6%)	37 (21.4%)
≥50%	52 (100.0%)	42 (80.8%)	10 (19.2%)
Cervical involvement	32 (100.0%)	27 (84.4%)	5 (15.6%)
Tumor size			
<2 cm	115 (100.0%)	105 (91.3%)	10 (8.7%)
≥2 cm	110 (100.0%)	98 (89.1%)	12 (10.9%)
Tumor localization			
Uterine fundus	192 (100.0%)	176 (91.7%)	16 (8.3%)
Lower uterine segment	16 (100.0%)	14 (87.5%)	2 (12.5%)
Uterine fundus + lower segment	17 (100.0%)	13 (76.5%)	4 (23.5%)
FIGO 2009 stage			
IA	153 (100.0%)	119 (77.8%)	34 (22.2%)
IB	23 (100.0%)	18 (78.3%)	5 (21.7%)
II	19 (100.0%)	16 (84.2%)	3 (15.8%)
IIIA	8 (100.0%)	5 (62.5%)	3 (37.5%)
IIIB	3 (100.0%)	1 (33.3%)	2 (66.7%)
IIIC	19 (100.0%)	19 (100.0%)	0 (0.0%)
FIGO 2009 stage			
Local disease (IA to II)	195 (100.0%)	153 (78.5%)	42 (21.5%)
Advanced disease (IIIA to IIIC)	30 (100.0%)	25 (83.3%)	5 (16.7%)
LVSI			
No	144 (100.0%)	112 (77.8%)	32 (22.2%)
Focal	45 (100.0%)	35 (77.8%)	10 (22.2%)
Substantial	36 (100.0%)	31 (86.1%)	5 (13.9%)
MELF	33 (100.0%)	26 (78.8%)	7 (21.2%)
Uterine length			
Mean (SD)	84 (18)	84 (17)	82 (23)
Median (IQR)	80 (70–90)	80 (70–90)	80 (70–90)
Myoma	104 (100.0%)	75 (72.1%)	29 (27.9%)
Adenomyosis	50 (100.0%)	36 (72.0%)	14 (28.0%)
Molecular classification			
Unknown	66 (100.0%)	46 (69.7%)	20 (30.3%)
POLEmut	2 (100.0%)	2 (100.0%)	0 (0.0%)
MMRd	54 (100.0%)	45 (83.3%)	9 (16.7%)
NSMP	89 (100.0%)	76 (85.4%)	13 (14.6%)
p53mut	14 (100.0%)	9 (64.3%)	5 (35.7%)
Prognostic risk group			
Low	126 (100.0%)	99 (78.6%)	27 (21.4%)
Intermediate	31 (100.0%)	24 (77.4%)	7 (22.6%)
High‐intermediate	30 (100.0%)	24 (80.0%)	6 (20.0%)
High	38 (100.0%)	31 (81.6%)	7 (18.4%)

*Note*: Values are expressed as number (percentage) unless otherwise indicated.

Abbreviations: BMI, body mass index; FIGO, International Federation of Gynecology & Obstetrics; IQR, interquartile range; LVSI, lymphovascular space invasion; MELF, microcystic, elongated, and fragmented; MMRd, mismatch repair deficient; NSMP, nonspecific molecular profile; p53mut, p53 mutated; POLEmut, polymerase‐ε‐mutated/ultramutated; SD, standard deviation.

Most of the surgeries were laparoscopic (90%) and performed by advanced surgeons (80%). The median surgery length was 115 min and blood loss was 50 mL. Adhesiolysis was necessary in 28% of cases. Severe perioperative complications occurred in <2% of cases (major vessel injury, excessive bleeding requiring conversion to laparotomy, nerve injury, and ureter injury). Postoperative complications in <1%, with two cases of lymphocyst—one following SNB and the other after side‐specific pelvic lymphadenectomy.

The overall detection rate was 90%. Bilateral success was achieved in 178 cases (79%), while unilateral success was observed in 25 cases (11%). Mapping failure was observed in 22 cases (10%).

The median number of removed SLNs in bilaterally successful procedures was three nodes. The final lymph node status was as follows: 170 negative, 14 isolated tumor cells, 12 micrometastases, and seven macrometastases. Five side‐specific pelvic lymphadenectomies were oerformed in preoperatively high‐intermediate and high‐risk cases with SNB failure. Overall, 22 cases lacked lymph node staging.

### 3.1 Bilateral or unilateral failure

Considering both bilateral and unilateral failed mapping together (*N* = 47, at least unilateral failures), higher BMI (*P* = 0.036), advanced FIGO 2009 stage (*P* = 0.019), and the presence of a myoma (*P* = 0.017) were the only statistically significant factors for mapping failure in univariable analysis. Other clinical and histopathological characteristics had minimal or no influence (Table 2).

**TABLE 2:** Univariable analysis: Bilateral or unilateral failure.

Characteristic	No. of patients	No. of failures	OR	95% CI	*P* value
Surgeon's experience					
Advanced surgeon	179	39	–	–	0.506
Trainee	46	8	0.76	0.31–1.68	
Age (years)	225	47	1.03	0.99–1.06	0.141
BMI	225	47	1.06	1.00–1.11	**0.036**
Cone biopsy					
No	212	45	–	–	0.603
Yes	13	2	0.67	0.10–2.63	
Myomectomy					
No	223	47	–	–	0.332
Yes	2	0	0.00		
Cesarean section					
No	201	40	–	–	0.309
Yes	24	7	1.66	0.61–4.13	
Adnexal surgery					
No	207	43	–	–	0.885
Yes	18	4	1.09	0.30–3.22	
Previous chemotherapy					
No	222	46	–	–	0.614
Yes	3	1	1.91	0.09–20.4	
Histotype					
Endometrioid (including mucinous)	211	43	–	–	0.481
Nonendometrioid	14	4	1.56	0.41–4.92	
Grade					
Low grade (grade 1 or 2)	189	40	–	–	0.815
High grade (grade 3)	36	7	0.90	0.34–2.10	
Myometrium infiltration					
<50%	173	37	–	–	0.735
≥50%	52	10	0.88	0.38–1.85	
Cervical involvement					
No	193	42	–	–	0.415
Yes	32	5	0.67	0.22–1.70	
Tumor size					
<2 cm	115	22	–	–	0.507
≥2 cm	110	25	1.24	0.65–2.38	
Tumor localization					
Uterine fundus	192	36	–	–	0.118
Lower uterine segment	16	4	1.44	0.39–4.42	
Uterine fundus + lower segment	17	7	3.03	1.04–8.45	
FIGO 2009 stage					
IA	153	34	–	–	**0.019**
IB	23	5	0.97	0.30–2.65	
II	19	3	0.66	0.15–2.12	
IIIA	8	3	2.10	0.41–9.00	
IIIB	3	2	7.00	0.65–153	
IIIC	19	0	0.00		
FIGO 2009 stage					
Local disease (IA to II)	195	42	–	–	0.532
Advanced disease (IIIA to IIIC)	30	5	0.73	0.23–1.88	
LVSI					
No	144	32	–	–	0.503
Focal	45	10	1.00	0.43–2.18	
Substantial	36	5	0.56	0.18–1.46	
MELF					
No	192	40	–	–	0.961
Yes	33	7	1.02	0.39–2.42	
Uterine length	225	47	1.0	0.98–1.01	0.566
Myoma					
No	121	18	–	–	**0.017**
Yes	104	29	2.21	1.15–4.34	
Adenomyosis					
No	175	33	–	–	0.171
Yes	50	14	1.67	0.79–3.41	
Molecular classification					
NSMP	89	13	–	–	0.267
MMRd	54	9	1.17	0.45–2.93	
p53mut	14	5	3.25	0.88–11.1	
POLEmut	2	0	0.00		
Prognostic risk group					
Low	126	27	–	–	0.972
Intermediate	31	7	1.07	0.39–2.65	
High‐intermediate	30	6	0.92	0.31–2.35	
High	38	7	0.83	0.31–2.00	
Approach					
Laparoscopy	202	39	–	–	0.102
Laparotomy	23	8	2.23	0.85–5.52	
Adhesiolysis					
No	163	32	–	–	0.457
Yes	62	15	1.31	0.64–2.59	

*Note*: Bold values represent significance of *p* < 0.05.

Abbreviations: BMI, body mass index; CI, confidence interval; FIGO, International Federation of Gynecology & Obstetrics; LVSI, lymphovascular space invasion; MELF, microcystic, elongated, and fragmented; MMRd, mismatch repair deficient; NSMP, nonspecific molecular profile; OR, odds ratio; p53mut, p53 mutated; POLEmut, polymerase‐ε‐mutated/ultramutated.

However, when multivariable logistic regression analysis was applied, only the presence of a myoma retained statistical significance (OR, 2.08 [95% CI, 1.08–4.11], *P* = 0.031), while the others became statistically insignificant (Table 3).

**TABLE 3:** Multivariable analysis: Bilateral or unilateral failure.

Characteristic	OR	95% CI	*P* value
BMI	1.05	1.00–1.11	0.067
Myoma			
No	–	–	0.031
Yes	2.08	1.08–4.11	

Abbreviations: BMI, body mass index; CI, confidence interval; OR, odds ratio.

### 3.2 Bilateral failure only

Regarding bilateral SNB failures (*N* = 22), univariable analysis identified BMI (*P* = 0.021) and FIGO 2009 stage (*P* = 0.046) as significant factors affecting success (Table 4). Interestingly, multivariable logistic regression analysis revealed that, in addition to BMI (OR, 1.11 [95% CI, 1.03–1.2], *P* = 0.007), age (OR, 1.09 [95% CI, 1.03–1.17], *P* = 0.008) was also an independent predictor of bilateral SNB failure (Table 5).

**TABLE 4:** Univariable analysis: Bilateral failure only.

Characteristic	No. of patients	No. of bilateral failures	OR	95% CI	*P* value
Surgeon's experience					
Advanced surgeon	179	20	–	–	0.131
Trainee	46	2	0.36	0.06–1.30	
Age (years)	225	22	1.05	1.00–1.10	0.064
BMI	225	22	1.08	1.01–1.17	**0.021**
Cone biopsy					
No	212	22	–	–	0.097
Yes	13	0	0.00		
Myomectomy					
No	223	22	–	–	0.520
Yes	2	0	0.00		
Cesarean section					
No	201	18	–	–	0.264
Yes	24	4	2.03	0.55–6.12	
Adnexal surgery					
No	207	21	–	–	0.500
Yes	18	1	0.52	0.03–2.75	
Previous chemotherapy					
No	222	21	–	–	0.260
Yes	3	1	4.79	0.22–52.0	
Histotype					
Endometrioid (incl. mucinous)	211	21	–	–	0.721
Non‐endometrioid	14	1	0.70	0.04–3.77	
Grade					
Low grade (grade 1 or 2)	189	18	–	–	0.772
High grade (grade 3)	36	4	1.19	0.33–3.44	
Myometrial infiltration					
<50%	173	18	–	–	0.554
≥50%	52	4	0.72	0.20–2.03	
Cervical involvement					
No	193	20	–	–	0.444
Yes	32	2	0.58	0.09–2.12	
Tumor size					
<2 cm	115	10	–	–	0.576
≥2 cm	110	12	1.29	0.53–3.17	
Tumor localization					
Uterine fundus	192	16	–	–	0.190
Lower uterine segment	16	2	1.57	0.23–6.32	
Uterine fundus + lower segment	17	4	3.38	0.88–10.9	
FIGO 2009 stage					
IA	153	16	–	–	**0.046**
IB	23	4	1.80	0.48–5.55	
II	19	0	0.00		
IIIA	8	2	2.85	0.40–13.6	
IIIB	3	0	0.00		
IIIC	19	0	0.00		
FIGO 2009 stage					
Local disease (IA or II)	195	20	–	–	0.519
Advanced disease (IIIA or IIIC)	30	2	0.63	0.10–2.31	
LVSI					
No	144	16	–	–	0.632
Focal	45	3	0.57	0.13–1.82	
Substantial	36	3	0.73	0.16–2.35	
MELF					
No	192	18	–	–	0.633
Yes	33	4	1.33	0.37–3.89	
Uterine length	225	22	1.01	0.99–1.04	0.226
Myoma					
No	121	9	–	–	0.203
Yes	104	13	1.78	0.73–4.49	
Adenomyosis					
No	175	14	–	–	0.111
Yes	50	8	2.19	0.83–5.47	
Molecular classification					
NSMP	89	6	–	–	0.646
MMRd	54	6	1.73	0.51–5.82	
P53mut	14	2	2.31	0.31–11.4	
POLEmut	2	0	0.00		
Prognostic risk group					
Low	126	12	–	–	0.921
Intermediate	31	4	1.41	0.37–4.41	
High‐intermediate	30	3	1.06	0.23–3.61	
High	38	3	0.81	0.18–2.74	
Approach					
Laparoscopy	202	18	–	–	0.231
Laprotomy	23	4	2.15	0.58–6.51	
Adhesiolysis					
No	163	15	–	–	0.642
Yes	62	7	1.26	0.46–3.15	

*Note*: Bold values represent significance of *p* < 0.05.

Abbreviations: BMI, body mass index; CI, confidence interval; FIGO, International Federation of Gynecology & Obstetrics; LVSI, lymphovascular space invasion; MELF, microcystic, elongated, and fragmented; MMRd, mismatch repair deficient; NSMP, nonspecific molecular profile; OR, odds ratio; p53mut, p53 mutated; POLEmut, Polymerase‐ε‐mutated/ultramutated.

**TABLE 5:** Multivariable analysis: Bilateral failure only.

Characteristic	OR	95% CI	*P* value
Age (years)	1.09	1.03–1.17	**0.008**
BMI	1.11	1.03–1.20	**0.007**

*Note*: Bold values represent significance of *p* < 0.05.

Abbreviations: BMI, body mass index; CI, confidence interval; OR, odds ratio.

## 4 DISCUSSION

Currently, SLN biopsy using intracervical ICG injection is a standard procedure in EC surgical staging. However, the reasons for failed mapping are still not satisfactorily defined, because of the heterogeneity of the published studies' design and results. The most significant risk factors for bilateral failure in our analysis of 225 surgeries were BMI and age.

Our overall (90%) and bilateral (79%) detection rate was similar to other studies, despite including all possible failure cases (failed ICG migration, diffuse smearing of the ICG, and no evidence of nodal tissue on final pathology) and not excluding initial SNB attempts as part of the learning curve of each surgeon. Some of these were omitted in other studies, which could have affected their success rate.^8^, ^11^

Obesity is one of the most debated potential risk factors for SNB failure. Eriksson et al. studied 472 patients undergoing robotic surgery using ICG or blue dye and demonstrated that successful mapping decreases with increasing BMI (irrespective of the dye used).^8^ However, they did not identify a critical BMI cutoff at which the mapping rate changes abruptly. Nevertheless, this finding has not been confirmed in other series.^11^, ^12^ In our study, the higher BMI significantly affected only bilateral mapping success in both univariable and multivariable analyses. This may support the theory that increasing BMI could modify the physiological pelvic lymphatic drainage by increasing vascular permeability and causing interstitial edema^13^ or simply make it more difficult to visualize lymphatic tissue due to the increased visceral adipose tissue.^8^

A similar hypothesis of increasing vascular permeability is considered regarding older age.^12^ In our cohort, age was a significant risk factor for bilateral mapping failure in multivariable analysis. Every additional year was associated with a 10% higher risk of failure. However, together with unilateral failure, age became statistically insignificant. This suggests that age may affect lymphatic drainage systematically. Nonetheless, other authors have not confirmed age as a significant factor.^11^, ^12^, ^14^

Considering possible local factors influencing mapping success, the presence of a myoma (*n* = 104, 46% of cases) was an interesting finding in our study. Using univariable analysis, it was a significant predictor (*P* = 0.017) of overall failure and remained significant in multiple regression analysis (*P* = 0.031). Similar results were published by Sozzi et al.^15^ Depending on the localization and size of the myoma, it may influence the physiological lymphatic uterine drainage; however, further investigations are necessary to confirm this theory. Histopathological characteristics (histology type, grade, LVSI, molecular classification) had minimal or no influence in our cases, in contrast with the study mentioned above. Using multivariate analysis, the study by Sozzi et al. identified LVSI (OR, 2.4 [95% CI, 1.04–1.12], *P* = 0.003) and nonendometrioid histology (OR, 3.0 [95% CI, 1.43–6.29], *P* = 0.004) as independent predictors of mapping failure.^15^ Nevertheless, other studies have confirmed our results and report these factors as insignificant.^11^, ^12^, ^14^, ^16^

Although larger tumors, those with deep myometrial invasion, or tumors extending into the lower uterine segment or cervical stroma could potentially alter lymphatic drainage patterns, leading to atypical sentinel node localization or mapping failure, this was not observed in our study, nor in others.^11^, ^12^, ^14^ On the other hand, in a prospective study in which different methods of sentinel node mapping (ultrasound‐guided myometrial injection of radiotracer) were applied, a tumor size <2 cm was associated with a higher SLN preoperative detection rate.^17^ Other studies focusing on factors that affect sentinel node localization are needed to explain this theory in more detail.

Advanced FIGO stage (III or IV) and enlarged lymph nodes are the most important risk factors for SNL mapping failure, as previously reported.^11^, ^15^, ^16^ A possible explanation for this is blocked lymphatic flow and the presence of tumorous thrombi in cases with high‐volume metastases.^11^, ^14^ Nevertheless, we did not prove this in our current study, as we focused on preoperative staging to exclude all cases with bulky lymph nodes and distant metastases, fully adhering to the European guidelines for SNB indications.^1^ As a result, there was no association between positive lymph node status and mapping failure. Even in the case of macrometastasis, all seven were successfully identified and removed. Additionally, there was no FIGO IV stage in the final pathological statement. In conclusion, ICG sentinel node mapping works well even in nodes with macrometastases under conditions where enlarged lymph nodes are detected preoperatively and excluded.

Previous pelvic surgeries (adnexal surgery: *n* = 18 [8%]; myomectomy: *n* = 2 [1%]; cesarean section: *n* = 24 [11%]) had no impact on SLN mapping in our cohort as well as in other series.^11^, ^12^ However, the main reason could be the absence of previous retroperitoneal surgeries in our cases, in which the surgical dissection of the parametria could damage the physiological lymphatic drainage.^12^ This might be an important factor in the future given the increasing number of patients undergoing excessive surgeries due to deep infiltrative endometriosis. Another potential risk factor not proven in our study was lysis of adhesions. To eliminate the potential adverse effect of adhesiolysis, this procedure should be performed at the very beginning of surgery, before ICG injection.^11^ Consequently, the patient's medical history is an integral part of surgery planning—not only the anamnesis of previous pelvic operations but also any history of previous abdominal inflammatory diseases are crucial factors.

Regarding the surgical procedure, we used cervical grasping by uterine manipulator immediately after the injection of ICG, except in cases with suspicious cervical tumor infiltration. According to a previous study, this practice has no impact on SLN detection.^17^ Additionally, we avoided another risk factor by using an ICG dose of 4–6 mL and performing reinjection in cases of failed ICG migration. It has been shown that an ICG dose <3 mL is associated with a higher risk of mapping failure.^9^

We consider the strengths of our study to be its prospective design and uniform surgical procedure. All surgeons followed the same application protocol to minimize the surgeon's influence, which was the only significant parameter for failure described by Ianieri et al.^12^ Nevertheless, those authors acknowledged that, although all surgeons were experts in the field of gynecologic oncology, the ICG procedure was not always performed by the same surgeon, but rather by several gynecologic oncology fellows.

The study's limitations include its single‐center design and nonrandomized allocation of cases to surgeons, which followed clinical practice. Presuming that more difficult surgeries (especially those involving patients who were morbidly obese or with a history of repeated surgical interventions) were predominantly assigned to more experienced surgeons, this resulted in a higher detection rate in the trainees' group, although this difference was not statistically significant.

## 5 CONCLUSIONS

In our single‐institution study, we showed that higher BMI and age are statistically significant factors for bilateral mapping failure. Additionally, the presence of a myoma was a significant local risk factor for overall (unilateral or bilateral) failure. Precise preoperative staging with the exclusion of inappropriate cases (bulky lymphadenopathy, distant metastases) is a crucial factor for SNB success. For surgeons, it is important to eliminate potentially influenceable factors by proper application technique (ICG >3 mL), adequate timing of individual surgical steps (adhesiolysis, opening retroperitoneal spaces), and careful visual or gentle palpation control of removed tissue. Other studies, ideally multicentric, are needed to validate our findings and help understand other potential risk factors for failure, which could be eliminated and could further improve bilateral SLN mapping outcomes.

## AUTHOR CONTRIBUTIONS

Petra Bretová: Conceptualization, data curation, project administration, writing – original draft. Luboš Minář: Conceptualization, methodology, supervision, writing – original draft. Petra Ovesná: Data curation, formal analysis, software, writing – review and editing. Vít Weinberger: Methodology, supervision, writing – review and editing. Michal Felsinger: Methodology, supervision, writing – review and editing. Michaela Koblížková: Data curation, writing – review and editing. Jitka Hausnerová: Investigation, methodology, writing – review and editing. Eva Jandáková: Investigation, methodology, writing – review and editing. Tatiana Stupková: Investigation, methodology, writing – review and editing.

## FUNDING INFORMATION

This work was supported by the Ministry of Health of the Czech Republic – RVO (FNBr 65269705).

## CONFLICT OF INTEREST STATEMENT

The authors declare no conflicts of interest.

## Data Availability

The data that support the findings of this study are available from the corresponding author upon reasonable request.
